# Comparison of Orthodontic Bracket Debonding Force and Bracket Failure Pattern on Different Teeth In Vivo by a Prototype Debonding Device

**DOI:** 10.1155/2021/6663683

**Published:** 2021-04-17

**Authors:** Tamzid Ahmed, Norma Ab Rahman, Mohammad Khursheed Alam

**Affiliations:** ^1^Department of Science of Dental Materials, Bangladesh Dental College, Dhaka, Bangladesh; ^2^Orthodontics Unit, School of Dental Sciences, Universiti Sains Malaysia, Kota Bharu, Kelantan, Malaysia; ^3^Orthodontic Division, Preventive Dentistry Department, College of Dentistry, Jouf University, Sakaka, Saudi Arabia

## Abstract

**Objective:**

To compare the orthodontic bracket debonding force and assess the bracket failure pattern clinically between different teeth by a validated prototype debonding device. *Materials and Method*. Thirteen (13) patients at the end of comprehensive fixed orthodontic treatment, awaiting for bracket removal, were selected from the list. A total of 260 brackets from the central incisor to the second premolar in both jaws were debonded by a single clinician using a validated prototype debonding device equipped with a force sensitive resistor (FSR). Mean bracket debonding forces were specified to ten (10) groups of teeth. Following debonding, Intraoral microphotographs of the teeth were taken by the same clinician to assess the bracket failure pattern using a 4-point scale of adhesive remnant index (ARI). Statistical analysis included one-way ANOVA with post hoc Tukey HSD and independent sample *t*-test to compare in vivo bracket debonding force, Cohen's kappa (*κ*), and a nonparametric Kruskal-Wallis test for the reliability and the assessment of ARI scoring.

**Results:**

A significant difference (*p* < 0.001) of mean debonding force was found between different types of teeth in vivo. Clinically, ARI scores were not significantly different (*p* = 0.921) between different groups, but overall higher scores were predominant.

**Conclusion:**

Bracket debonding force should be measured on the same tooth from the same arch as the significant difference of mean debonding force exists between similar teeth of the upper and lower arches. The insignificant bracket failure pattern with higher ARI scores confirms less enamel damage irrespective of tooth types.

## 1. Introduction

Fixed orthodontic treatment outcomes rely much on the integrity of the orthodontic bracket-adhesive system. To resist accidental bracket failures, many studies focused on the bonding ability of the various orthodontic adhesives, brackets, surface conditioning, and bonding methods. These studies are either in vitro or ex vivo done in the laboratory for mechanical testing or in vivo analysis of clinical bracket failure rates. The mechanical tests are usually done by the universal testing machine that applies either true shear or tensile load on the brackets. The machine is known for its accuracy and precision. But, it cannot exactly imitate the mechanism of clinical debonding, as the brackets face combined forces in all directions while functioning and clinical debonding [1]. Besides, the machine impacts at a much lower velocity in comparison to the clinical debonding [2]. Therefore, the materials should be tested in the atmosphere and under certain conditions where it is intended to function. Aging or biodegradation of the orthodontic bonding system has a negative influence on its bonding efficiency. This supports the evidence of lower bracket bond strength clinically [3–6]. Studying clinical bracket failure rates inside a controlled atmosphere of the oral cavity is laborious and requires to prolong monitoring. Also, the universal testing machine cannot be introduced clinically due to its large dimensions. Therefore, a conventional manufacturer-made plier capable of measuring the force while debonding brackets simultaneously in a standardized manner was recommended [2]. Devices introduced so far for measuring the clinical bracket debonding force are either made of digital force gauge (DFG) or strain gauge [3, 5, 7–11]. The DFG-equipped devices were elastic spacer instrument modified purposefully for debonding, not a regular debonding plier. On the contrary, the strain gauge relies solely on the elastic straining of the plier handles. Besides, some of the devices were not validated and some of them were not applied clinically [7, 9–11]. Bracket failure patterns by some devices are unknown which might risk enamel damage [3, 6, 8].

Orthodontic bracket bonding specific to the tooth types was studied rarely in vitro [12–14]. In contrast, most of the studies considered orthodontic bracket bond strength on the premolars as representative of all tooth types. Some considered the similar teeth of both arches as one group [1, 15]. Only one study measured orthodontic bracket bond strength in vivo on distinct tooth types but limited to the upper arch [8].

Therefore, this clinical experiment was intended to compare the orthodontic bracket debonding force between distinctive tooth types by a “novel method” utilizing a prototype with a force sensor. Following debonding, the clinical bracket failure pattern on each tooth type was also assessed to justify the use of this new device in terms of enamel damage.

## 2. Materials and Method

### 2.1. Sample Size

Orthodontic bracket debonding force was recorded from 260 teeth, ranging from the incisors up to the premolars in thirteen (13) patients. This exact sample size of 260 was calculated by the G∗Power software, version 3.1, with the power of 80% and alpha error probability of 0.05 [16].

### 2.2. Sample Collection

All the subjects were at the stage of termination of the extensive fixed orthodontic therapy awaiting bracket debonding. They were chosen from the waiting list of the orthodontic outpatient department. The subjects were inquired of a comprehensive medical and dental history followed by a thorough dental examination. The inclusion criteria of the selection were the subject's approval of participating in the study, treatment with traditional metallic brackets, and the intact dentition in both upper and lower arches. Any sample with the presence of cracks, decay, or any other deformities and anomalies; the presence of any dental restoration; and enamel surface preparation using alcohol, formalin, hydrogen peroxide, etc. were excluded. A written consent including the detailed study procedure was provided and signed by each subject upon acknowledging the conditions and the study method.

### 2.3. Sample Preparation

The selected individuals were averagely aged 24 ± 5.2 years. 0.022^″^ EPS (elite performance series) metallic brackets (MEM Dental Technology, Tainan City, Taiwan) were bonded using Transbond XT adhesive (3M Unitec, Monrovia, California, USA). The bonding procedure was performed by the same clinicians maintaining a strict protocol. Before bonding, scaling followed by polishing by a slurry of pumice was done. Surface conditioning was done for 15 seconds for each tooth and then gently air-blown for uniform dispersion of liquid. Orthodontic adhesives were polymerized by LED light activation (model DB686, COXO, Guangdong, China). The total duration of curing was 20 seconds for each tooth, divided equally into 10 seconds for covering the mesial and distal side. The mean interim period between the bonding and debonding was 25 ± 9 months.

### 2.4. Bracket Debonding

**Figure 1:**
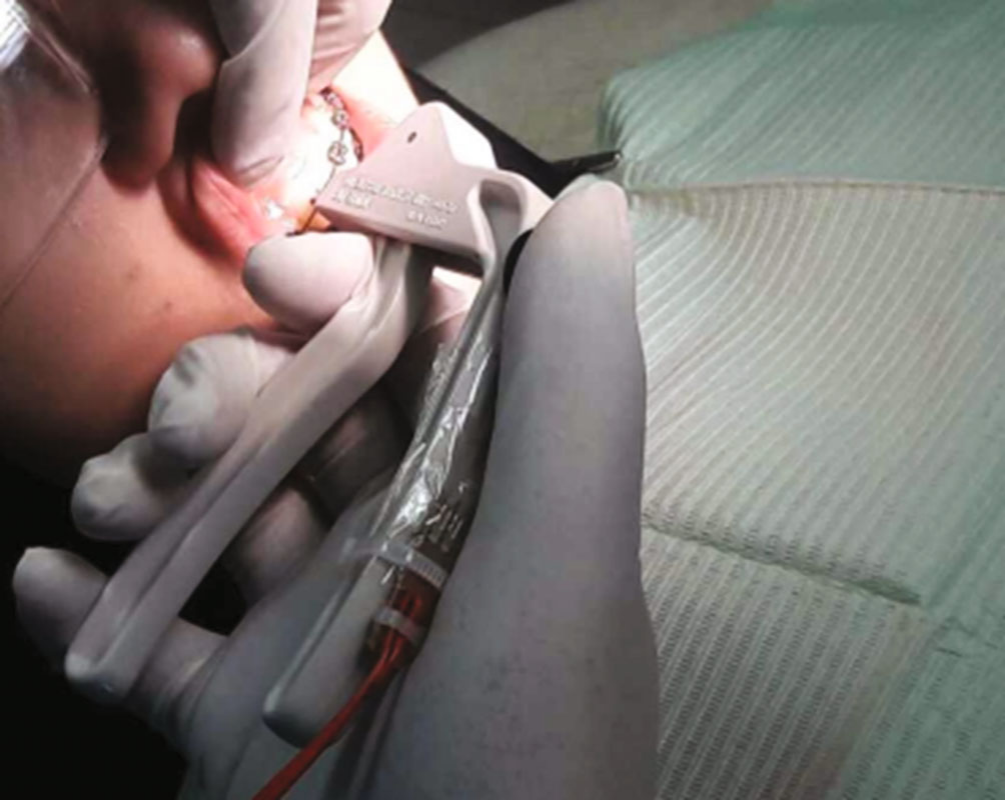
In vivo orthodontic bracket debonding by the prototype.

A prototype constructed of a manufacturer-made debonding plier (i.e., lift-off debonding instrument) (3M Unitec, Monrovia, California, USA) and a force sensor (i.e., FSR) (Model: 402, Interlink, California, USA) was introduced to debond all the brackets from both sides of the incisors to the premolars in both jaws. The force sensor was calibrated and then attached to the posterior fixed handle of the plier using a cable tie and adhesive tape. The mechanism of the prototype with its validity and reliability was stated previously [17]. The same clinician debonded all the brackets holding in a standardized manner (Figure 1). The bracket fixing wire loop of the prototype was clamped on a bracket wing, and then, the plier's arms were squeezed by compressing the central area of the force sensor by the thumb until debonding. The maximum force exerted at the incident of bracket failure was recorded by the force sensor and interpreted into the newton (N) units of force [17].

### 2.5. Assessment of Clinical Bracket Failure Pattern

**Figure 2:**
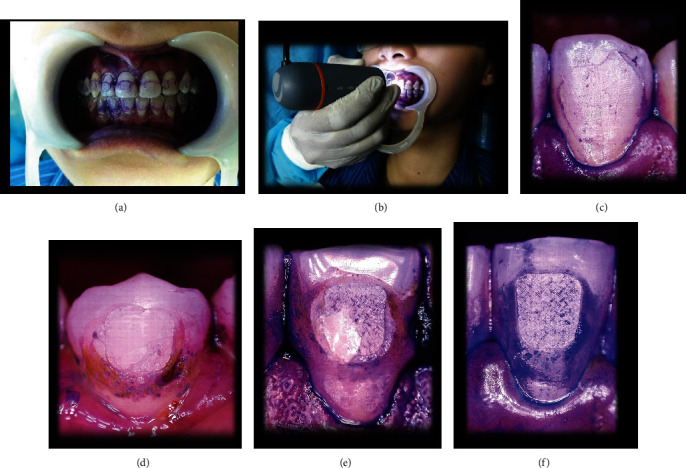
Assessment of in vivo ARI. (a) Application of disclosing medium. (b) Microphotographs took using a portable digital microscope. (c) Score 0: no adhesive on enamel. (d) Score 1: less than half adhesive on enamel. (e) Score 2: more than half adhesive on enamel. (f) Score 3: all the adhesive on enamel with distinct impression of bracket.

On completion of the debonding process, the loose adhesive fragments were rinsed off and the subjects were instructed to chew a disclosing tablet (i-C2, Ortho-Care Ltd., West Yorkshire, UK). This enhanced the contrast between the enamel surface and the adhesive (Figure 2). A calibrated portable digital microscope (Celestron, California, USA) was used to assess the ARI in vivo. The ARI scores were ranged between 0 and 3 as described by Årtun and Bergland [18]. For calibration and image analysis, the microscope was supported by software (Celestron Micro Capture Pro, Version 1.0, Celestron, California, USA). The magnification was fixed at 30x. Standardized microscopic photographs were captured highlighting the whole tooth surface specifically the remaining adhesive (Figure 2). The photographs were processed at the resolution of 2560 × 1920 pixels, 24-bit color depth, and formatted as JPEG (Joint Photographic Experts Group) files. For reliability, ARI scoring was done twice separately by two different raters.

### 2.6. Ethical Consideration

Ethical approval of conducting the study was obtained from the Human Research and Ethics Committee (JEPeM) of the Universiti Sains Malaysia (study protocol code: USM/JEPeM/17020075).

### 2.7. Statistical Analysis

Statistical data analyses were performed by the SPSS software (version 24.0, Armonk, New York, USA) with the significance level (*p* < 0.05). A one-sample Kolmogorov-Smirnov test was done to assess the normality of all data distribution. For the comparison of mean debonding force between different tooth groups, one-way analysis of variance (ANOVA) was done with a post hoc Tukey HSD test. Besides, orthodontic bracket bond strength—between the upper and lower teeth and between the anterior and posterior teeth—was comparatively analyzed utilizing independent sample *t*-tests. The nonparametric Kruskal-Wallis test was done for assessing the ARI. The reliability of the ARI scoring was investigated by Cohen's kappa (*κ*) statistics.

## 3. Results

The descriptive statistics of the debonding force with one-way ANOVA results are listed in Tables 1 and 2. The mean debonding forces were significantly (*p* < 0.001) different according to the tooth types. Multiple pairwise group comparisons revealed the significant difference of upper second premolars to all groups except the lower central incisors and the upper first premolars. However, in maxillary dentition, the anterior teeth had greater bond strength (*p* < 0.001) in comparison to the posterior teeth (Table 3). Conversely in the lower dentition, the bracket bond strength was significantly (*p* = 0.012) greater in the posterior teeth (Table 3). When the upper and lower teeth as a whole compared, there was no significance (*p* = 0.284). But the mean bracket debonding force was significantly higher (*p* < 0.001) in lower premolars in comparison to the upper premolars when the similar teeth of both arches were compared individually (Table 4).

**Figure 3:**
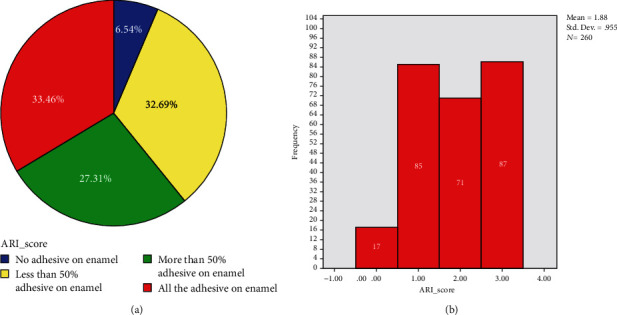
Overall frequency and distribution (percentage) of ARI scores in all tooth groups.

For the reliability of ARI scoring, almost prefect agreement was confirmed (*k* = 0.841, *p* < 0.001) between the raters. The distribution of ARI scoring was similar (*p* = 0.921) among all tooth groups (Table 5). Of a total of 226 samples, score 3 (*n* = 87, 33.46%) was most frequent followed by score 1 (*n* = 85, 32.69%), score 2 (*n* = 71, 27.31%), and score 0 (*n* = 17, 6.54%) (Figure 3). Overall, the percentage of higher scores (i.e., scores 3 and 2) was dominant (60.77%) in comparison to that of the lower scores (i.e., scores 1 and 0).

## 4. Discussion

Commonly, orthodontic bracket bond strength is measured as average shear or tensile stress (i.e., MPa units), dividing the maximum force by the area of the bracket base. But in a finite element analysis, it was revealed that it is the maximum force that debonds brackets, not the average stress [1]. Therefore, the bracket bond strength is expressed as the maximum debonding force in Newton units.

Debonding force was measured in patients following a full course of fixed orthodontic therapy taking an average of 25 ± 9 months. Previously, in vivo bracket bond strength following a comprehensive treatment was also investigated but was particularly limited to the maxillary premolars [6]. Besides, the study protocol differs from the current study which makes the comparison of results impossible. The same applies to another study that measured the bracket debonding forces from the maxillary dentition [8]. Bracket debonding forces measured in this study are significantly (*p* < 0.001) different between different teeth (Table 1). Despite the difference in the methodology, this outcome is similar to that of the previous studies [8, 12, 14]. With further analysis, the results exhibited that in the upper arch, the bracket bond strength was higher significantly (*p* < 0.001) on the anterior teeth than on the posteriors (i.e., premolars), whereas in the lower segment, the bracket bonding strength on the posterior teeth (i.e., premolars) was significantly (*p* = 0.012) higher (Table 3). A similar kind of result was also observed in some of those studies mentioned earlier [8, 12, 14]. These findings suggest no distinct association between the debonding force and the etch pattern in both enamel and dentine as it was previously assumed that the prismless enamel which is more common in posterior teeth is related to the poor quality of etching and hence poor bonding [19].

In the case of self-etch adhesives, in addition to the micromechanical retention, chemical reaction takes place by the transfer of ions between functional monomers and calcium in the remaining hydroxyapatite of dentine [20]. The remaining hydroxyapatite crystals particularly the calcium ions which are crucial for bonding may vary with tooth types requiring further investigation.

On the comparison between the similar teeth of the upper and lower jaw, the upper and lower premolars had significantly (*p* < 0.01) different debonding force values which is identical to the previous in vitro findings. Therefore, to standardize the methodology, the orthodontic bracket bond strength should be studied on the same tooth samples either from the upper or lower jaw [12, 14].

Ideally, the debonding force should be of such a limit that successfully removes the bracket causing minimal iatrogenic loss of enamel. Most commonly, data collected from the in vitro studies testing bond strength of the orthodontic materials are inferred to the in vivo clinical situations. Previously, it was stated that the maximum orthodontic bracket bond strength should be 9.7 MPa to prevent enamel fracture [21]. Another study suggested the bond strength ranging between 5.9 and 7.8 MPa, which is clinically admissible [22]. Although the results are not reported as average stress (i.e., MPa units), it is easy to estimate that the mean debonding forces reported in this study are considerably lower than these established data. This is explainable as the pattern and area of the applied force in the current study differed alongside the testing condition. The device mainly exerts tensile force on the bracket wing with the combination of sheer-peel and torsional loads. In contrast, the universal testing machine applies either true shear force or tensile force to debond brackets. The debonding plier used in the study (i.e., LODI) requires less force in comparison to the traditional lab-based mechanical tensile force to debond brackets [23]. This lower level of force can be best explained by the finite element analysis as the LODI impacts with higher and uneven stress distribution within the structures of the bracket-adhesive-enamel [24]. According to Williams et al., 1000 grams of force which is analogous to 9.8 N is to be a suitable limit to apply directly to a tooth [25]. The mean bracket debonding forces were reported within this limit (Table 1). The actual range of in vitro bond strength applicable for the clinical situation is unclear as it is still a topic of argument [6].

Following debonding, the remaining adhesives on the teeth were examined and assessed by the ARI. This was done to analyze the device's likelihood of enamel damage relative to the tooth types. Bracket debonding that mostly involves bracket-adhesive interface causes less enamel damage [26, 27, 28]. The microscope's advantageous dimension allowed to take standardized photographs suitably from the incisors to the premolars in both arches. Photographs were taken by the same clinician keeping the microscope at a right angle to each tooth surface, specifically the remaining adhesive. The microscope was set at 30x magnification, as ARI evaluation by the naked eye and the most commonly practiced 10x magnification under the stereomicroscope were similar [29]. The difference in ARI scoring between different teeth was not significant (Table 5). This ensures the device's consistent pattern of bracket debonding irrespective of the tooth types. The result differed from a previous study due to the difference of the debonding method, as the ARI scoring varies with different debonding techniques [27].

ARI score of 3 was most frequently (33.46%) observed indicating brackets were debonded mostly at their interface with the adhesive, which is beneficial for preserving the surface enamel. Score 0 was least observed (6.54%). Moreover, the higher scores (i.e., scores 2 and 3) were more frequent (60.77%) than those of the lower scores (39.23%) (Figure 3). Therefore, the prototype device introduced can be considered for clinical application due to less enamel damage.

## 5. Conclusion

The study succeeded in reporting clinical data on orthodontic bracket debonding force with a favorable bracket failure pattern (i.e., less enamel damage) on different teeth with significant outcomes. As the bracket bond strength varies with tooth types, it is more logical to study on the same tooth samples either from the upper or lower arch.

## Figures and Tables

**Table 1:** Descriptive analysis of in vivo bracket debonding force values.

Groups	*n*	Mean ± standard deviation (Newton)
U1	26	9.46 ± 2.22
U2	26	9.57 ± 2.55
U3	26	9.65 ± 2.39
U4	26	7.94 ± 2.57
U5	26	7.03 ± 1.74
L1	26	8.18 ± 1.81
L2	26	8.92 ± 2.51
L3	26	8.92 ± 1.52
L4	26	9.30 ± 1.70
L5	26	9.81 ± 1.93

U1: upper central incisor; U2: upper lateral incisor; U3: upper canine; U4: upper first premolar; U5: upper second premolar; L1: lower central incisor; L2: lower lateral incisor; L3: lower canine; L4: lower first premolar; L5: lower second premolar.

**Table 2:** One-way ANOVA of orthodontic bracket debonding force in vivo.

Variable		Sum of squares	Mean square	*F*	*p* value
Debonding force (Newton)	Between groups	188.916	20.991	4.635	<0.001^∗^
	Within groups	1132.299	4.529		
	Total	1321.215			

**Table 3:** Comparison of orthodontic bracket debonding force between the anterior and posterior teeth in both jaws.

Variables	Mean ± standard deviation		*t* statistics	*p* value
Debonding force (Newton)	Upper anterior (*n* = 78)	Upper posterior (*n* = 52)	5.038	<0.001^∗^
	9.56 ± 2.36	7.48 ± 2.22		
	Lower anterior	Lower posterior	-2.545	0.012^∗^
	8.68 ± 1.99	9.56 ± 1.82		

^∗^ *p* < 0.05.

**Table 4:** Difference of mean debonding force between the upper and lower premolars.

Variable	Mean ± standard deviation		*t* statistics	*p* value
Debonding force (Newton)	Upper premolars (*n* = 52)	Lower premolars (*n* = 52)	-5.207	<0.001^∗^
	7.48 ± 2.22	9.56 ± 1.82		

^∗^ *p* < 0.05.

**Table 5:** ARI scoring between different tooth groups.

Variable	Chi-square	df	*p* value
ARI score	3.856	9	0.921

## Data Availability

Details are presented within the article in the form of tables and text in results. Other data will be made available upon request.

## References

[1] Katona T. R. (1997). A comparison of the stresses developed in tension, shear peel, and torsion strength testing of direct bonded orthodontic brackets. *American Journal of Orthodontics and Dentofacial Orthopedics*.

[2] Eliades T., Brantley W. A. (2000). The inappropriateness of conventional orthodontic bond strength assessment protocols. *European Journal of Orthodontics*.

[3] Hajrassie M. K. A., Khier S. E. (2007). In-vivo and in-vitro comparison of bond strengths of orthodontic brackets bonded to enamel and debonded at various times. *American Journal of Orthodontics and Dentofacial Orthopedics*.

[4] Murray S. D., Hobson R. S. (2003). Comparison of in vivo and in vitro shear bond strength. *American Journal of Orthodontics and Dentofacial Orthopedics*.

[5] Penido S. M., Penido C. V., dos Santos-Pinto A., Gandini L. G., Bagnato V. S. (2009). In vivo and in vitro study of the shear bond strength of brackets bonded to enamel using halogen or LED light. *World Journal of Orthodontics*.

[6] Pickett K. L., Lionel Sadowsky P., Jacobson A., Lacefield W. (2001). Orthodontic in vivo bond strength: comparison with in vitro results. *The Angle Orthodontist*.

[7] Brosh T., Kaufman A., Balabanovsky A., Vardimon A. D. (2005). In vivo debonding strength and enamel damage in two orthodontic debonding methods. *Journal of Biomechanics*.

[8] Hassan A. H. (2010). Shear bond strength of precoated orthodontic brackets: an in vivo study. *Clinical, Cosmetic and Investigational Dentistry*.

[9] Hildebrand N. K. S., Raboud D. W., Heo G., Nelson A. E., Major P. W. (2007). Argon laser vs conventional visible light-cured orthodontic bracket bonding: an in-vivo and in-vitro study. *American Journal of Orthodontics and Dentofacial Orthopedics*.

[10] Prietsch J. R., Spohr A. M., da Silva I. N. L., Beck J. C. P., Oshima H. M. S. (2007). Development of a device to measure bracket debonding force in vivo. *European Journal of Orthodontics*.

[11] Tonus J. L., Manfroi F. B., Borges G. A., Grigolo E. C., Helegda S., Spohr A. M. (2017). Prototype to measure bracket debonding force in vivo. *Dental Press J Orthod.*.

[12] Hobson R. S., McCabe J. F., Hogg S. D. (2001). Bond strength to surface enamel for different tooth types. *Dental Materials*.

[13] Linklater R. A., Gordon P. H. (2001). An ex vivo study to investigate bond strengths of different tooth types. *Journal of Orthodontics*.

[14] Öztürk B., Malkoç S., Koyutürk A. E., Çatalbaş B., Özer F. (2008). Influence of different tooth types on the bond strength of two orthodontic adhesive systems. *European Journal of Orthodontics*.

[15] Bishara S. E., Vonwald L., Zamtua J., Damon P. L. (1998). Effects of various methods of chlorhexidine application on shear bond strength. *American Journal of Orthodontics and Dentofacial Orthopedics*.

[16] Faul F., Erdfelder E., Lang A.-G., Buchner A. (2007). G∗ Power 3: a flexible statistical power analysis program for the social, behavioral, and biomedical sciences. *Behavior Research Methods*.

[17] Ahmed T., Rahman N. A., Alam M. K. (2019). Validation and reliability of a prototype orthodontic bracket debonding device equipped with force-sensitive resistor (FSR): a novel method of measuring orthodontic bracket debonding force in vivo. *Progress in Orthodontics*.

[18] Årtun J., Bergland S. (1984). Clinical trials with crystal growth conditioning as an alternative to acid- etch enamel pretreatment. *American Journal of Orthodontics*.

[19] Whittaker D. K. (1982). Structural variations in the surface zone of human tooth enamel observed by scanning electron microscopy. *Archives of Oral Biology*.

[20] Van Meerbeek B., De Munck J., Yoshida Y. (2003). Adhesion to enamel and dentin: current status and future challenges. *Operative Dentistry-University of Washington*.

[21] Retief D. H. (1973). Effect of conditioning the enamel surface with phosphoric acid. *Journal of Dental Research*.

[22] Reynolds I. R., von Fraunhofer J. A. (1976). Direct bonding of orthodontic brackets—a comparative study of adhesives. *British Journal of Orthodontics*.

[23] Parrish B. C., Katona T. R., Isikbay S. C., Stewart K. T., Kula K. S. (2012). The effects of application time of a self-etching primer and debonding methods on bracket bond strength. *The Angle Orthodontist*.

[24] Katona T. R., Moore B. K. (1994). The effects of load misalignment on tensile load testing of direct bonded orthodontic brackets--a finite element model. *American Journal of Orthodontics and Dentofacial Orthopedics*.

[25] Williams O. L., Bishara S. E., Ortho D. (1992). Patient discomfort levels at the time of debonding: a pilot study. *American Journal of Orthodontics and Dentofacial Orthopedics*.

[26] Pont H. B., Özcan M., Bagis B., Ren Y. (2010). Loss of surface enamel after bracket debonding: an in-vivo and ex-vivo evaluation. *American Journal of Orthodontics and Dentofacial Orthopedics*.

[27] Bonetti G. A., Zanarini M., Parenti S. I., Lattuca M., Marchionni S., Gatto M. R. (2011). Evaluation of enamel surfaces after bracket debonding: an in-vivo study with scanning electron microscopy. *American Journal of Orthodontics and Dentofacial Orthopedics*.

[28] Janiszewska-Olszowska J., Tandecka K., Szatkiewicz T., Sporniak-Tutak K., Grocholewicz K. (2014). Three-dimensional quantitative analysis of adhesive remnants and enamel loss resulting from debonding orthodontic molar tubes. *Head & Face Medicine*.

[29] Montasser M. A., Drummond J. L. (2009). Reliability of the adhesive remnant index score system with different magnifications. *The Angle Orthodontist*.

